# Express Analysis of Cartilage Tissue Using Multivariate Analysis of IR Spectra

**DOI:** 10.17691/stm2022.14.6.03

**Published:** 2022-11-28

**Authors:** N.Yu. Ignatieva, O.L. Zakharkina, A.P. Sviridov

**Affiliations:** Associate Professor, Department of Physical Chemistry; Lomonosov Moscow State University, 1 Leninskie Gory, Moscow, 119991, Russia;; Researcher, Institute of Photonic Technologies; Federal Scientific Research Centre “Crystallography and Photonics” of the Russian Academy of Sciences, 59 Leninsky Prospect, Moscow, 119333, Russia; Leading Researcher, Institute of Photonic Technologies; Federal Scientific Research Centre “Crystallography and Photonics” of the Russian Academy of Sciences, 59 Leninsky Prospect, Moscow, 119333, Russia

**Keywords:** cartilage tissue, collagen, glycosaminoglycans, IR spectroscopy with attenuated total reflection, principal component regression method

## Abstract

**The aim of the study** was to develop a diagnostic method for the quantitative determination of the main components of cartilage tissue of various types based on multivariate IR spectral analysis and verification of data using classical chemical analysis.

**Materials and Methods:**

Cartilages of the nasal septum, knee joint, rib, and nucleus pulposus of the intervertebral disc, as well as trypsinized and defective cartilage samples, were examined as samples. The IR spectra of the cartilage samples, as well as calibration mixtures of collagen and chondroitin sulfate, were obtained. The IR spectra were collected using the attenuated total reflectance techniques, and their processing was performed using the TQ Analyst software and the principal component regression calibration technique. Based on calibration dependence, the *K*_sp_ coefficient was determined as the ratio of the mass fractions of collagen and chondroitin sulfate. Its value was compared with the value of *K*_chem_, equal to the ratio of the mass fractions of collagen and chondroitin sulfate, obtained using the classical chemical analysis of these substances.

**Results:**

The IR spectra of cartilage tissues are a superposition of the IR spectra of collagen and chondroitin sulfate and qualitatively reflect their composition. A change in the ratio between the relative intensities of the characteristic bands of compounds in the IR spectrum is obvious only with a significant change in the content of these compounds in cartilage. This change occurs after trypsinization, when *K*_sp_ increases from 0.88±0.05 (*K*_chem_~0.8) to 4.55. The use of a calibration model with a complete analysis of the cartilage IR spectrum made it possible to determine the difference in the ratio of the main components in the matrix of different samples in the absence of obvious changes in the IR spectra. Thus, a statistically significant decrease in the content of chondroitin sulfate in degraded articular cartilage (*K*_sp_=4.4±1.8; *K*_chem_~5.5) was shown compared with intact samples (*K*_sp_=2.8±1.1; *K*_chem_~2.6).

**Conclusion:**

IR spectrometric express analysis of cartilage tissue employing the principal component regression method allows a correct determination of the ratio of the main components in the cartilage matrix, those of collagen and glycosaminoglycans. The proposed technique includes one measurement, does not require prolonged and laborious sample preparation, does not require long, multi-stage and laborious chemical manipulations to determine each of the components, and makes it possible to determine the features and changes in the composition for a large set of samples of cartilage tissue of different types. In future, this approach can be used for non-invasive diagnostics of cartilage tissue.

## Introduction

The main components of the matrix of cartilage tissues are collagen (structural protein) and sulfated polysaccharides — glycosaminoglycans (GAGs), which form complex compounds with protein (proteoglycans and their aggregates) [1, 2]. The content of these components and their ratio are one of the most important indicators of the functional ability of cartilage and cartilage materials [1, 2]. These indicators are estimated in the study of pathological changes [3-11] and zonal structure [12] of cartilage, as well as in the creation of their substitutes (scaffolds) [13]. A significant number of test samples is required to establish reliable correlations between the composition and characteristics of a particular type of cartilage material.

Classical chemical analysis of biological tissues usually requires multi-stage sample preparation, which includes obtaining soluble forms (the first stage) and the analysis of solutions for each of the components (the second stage). At the same time, the transfer of tissue into a soluble form cannot be performed using the same method for both collagen and GAGs, and the analysis of each component requires special analytical equipment and highly skilled personnel.

Multistage sample preparation and analysis, and, consequently, the use of expensive consumable reagents, can be excluded by measuring a set of physical values of an integral structure. In this case, the establishment of a relationship between the obtained set of physical values and chemical characteristics will remain challenging. These methods include IR spectroscopy. The theoretical basis for the use of IR spectroscopy for the analysis, interpretation, and mathematical processing of IR spectra of biological fluids and tissues is presented in the paper [14]. The paper [15] gives the analysis of blood data in which an original approach to the statistical processing of the ratios of absorption band peak heights has been described in detail, which allows drawing a conclusion about pathological changes in a biological system.

The usefulness of IR spectra of cartilage tissue to assess the content of the main components in it was discussed already more than 20 years ago [16]. The earlier studies showed at a qualitative level that after cartilage trypsinization (and removal of a significant part of GAGs from the matrix), the IR absorption spectrum in the region of 700–1700 cm^–1^ changes considerably [16, 17]. Characteristic bands related to individual components were identified, and the ratio of the peak intensities of these bands was used to estimate the content of the main components [13, 18, 19]. Unfortunately, this approach does not establish strict quantitative ratios between the components [12], although it gives the qualitative mapping of the GAG content in cartilage [18, 19].

The situation has changed with the use of formal methods of multivariate analysis of large complex arrays of experimental data (IR spectra of multicomponent systems) in IR spectroscopy. Thus, researchers, creating their own processing algorithms with the use of multivariate analysis of the IR spectra of cartilage materials, have reached a good accord on the amounts of GAGs determined by spectral and chemical methods of analysis in joint cartilage [11], nasal septum [12], cartilage scaffolds [13] and also assessed a degree of degradation of the articular cartilage [6]. The software packages for multivariate data analysis created in the software for serial devices greatly facilitate the quantitative determination of the two main components in the cartilage matrix. Nevertheless, to prove the reliability and accuracy of the method of IR spectral data analysis, it is necessary to compare the final outcome with the data obtained by traditional methods.

Attenuated total reflectance (ATR) spectroscopy is of particular interest. It provides performing express measurements of cartilage spectra using a prism tip, which is only in contact with the sample, which significantly simplifies sample preparation. However, only the near-surface layer of the sample, where the evanescent field penetrates during total reflection, participates in the spectrum measurements. It is clear that the IR spectrum of the surface layer of the sample may differ from the IR spectrum averaged over the volume. The question of the adequacy of determining the composition of cartilage by ATR IR spectroscopy is still open.

**The aim of the study** was to develop a method for diagnosing cartilage tissue of various types with a quantitative determination of the main components based on multivariate analysis of IR spectra and to verify the data using classical chemical analysis.

## Materials and Methods

### Materials

Porcine nasal septum and rib cartilage, as well as a cow knee joint and fragments of rabbit and sheep spines, were acquired from the farm and were pre-treated no later than 12 h after removal.

The samples were washed in a NaCl solution, and the fragments intended for the study were derived. For spectral and subsequent biochemical analyses, 0.5–0.8-mm thick plates with an area of approximately 0.5×0.5 cm^2^ were cut out from cartilage tissue. Such plates were derived from the middle part of the transverse sections of the nasal septum and costal cartilage. Samples of articular cartilage were taken separately from visually smooth areas and from rough areas with pronounced fibrousness. The nuclei pulposi were removed from the intervertebral discs. After isolation, the specimens were dried in a thermostat at 37°C in the air for 18 h, and their IR spectra were collected. Thereafter, each preparation was divided into equal parts, the GAG content was determined in one half of the sample, and the collagen content was determined in the other one.

One of the components of the calibration mixture was chondroitin-4-sulfate (ChS) (C6737; Merck, USA), one of the most common GAGs in cartilage tissue. Type I collagen (C3867; Sigma-Aldrich, USA) was used as the second component. A calibration solution with a mass ratio of collagen and ChS from 0.11 to 49.0 was placed on a glass slide and dried in a thermostat at 37°C for 18 to 24 h. As a result, a film was formed on the glass slide, which was used as a standard for obtaining the IR spectrum.

#### First trypsinization

Cartilage tissue specimens weighing 1.5–2.0 mg were placed in a solution containing trypsin at a concentration of 1 mg/ml and sodium azide at a concentration of 0.2 mg/ml, kept in a thermostat at 37°C for 24 h. Part of the sample material passed into the supernatant (C1), another part turned out to be undissolved (O1). In a separate experiment with cartilage of the nasal septum, the supernatant was decanted, dried in a glass Petri dish, and the IR spectrum of the obtained film was measured. The undissolved material was washed, dried and its IR spectrum was measured. In other cases, the entire system was subjected to heating and repeated trypsinization.

#### Second trypsinization

The supernatant and undissolved residue remaining after the first trypsinization were heated in a water bath at 85°C for 15–20 min, then trypsin was added to a final concentration of 2 mg/ml and left in a thermostat at 37°C for another 24 h. The resulting colloidal mixture was centrifuged, the supernatant (C2) was decanted. In a separate experiment, centrifugate (O2) and supernatant C2 were dried, and their IR spectra were collected. In all other cases, C2 from the second trypsinization was analyzed for GAG content using a spectrophotometric reaction.

### Methods

#### Determination of the glycosaminoglycan content

The amount of GAGs was determined in the supernatant after the second trypsinization of the dried samples. The assay is based on the complexation reaction of anionic GAGs with dimethylmethylene blue (DMMB) which is accompanied by metachromasia. The analysis was carried out according to the method proposed by Farndale et al. [20] with minor modifications, which made it possible to significantly reduce the standard deviation for calibration dependence. The changes consisted in the replacement of hydrochloric acid with acetic acid in the preparation of a DMMB solution, in the exclusion of adding NaCl to this solution, and in the additional processing of the dye solution in an ultrasonic bath. ChS solutions (C6737) at a concentration of 1–5 μg/ml were used as a calibration standard. The content of the ChS–DMMB complex was estimated by differential spectrophotometry at a wavelength of 589 nm using a UV-1800 spectrophotometer (Shimadzu Co., Japan) and a DMMB solution as the reference solution. Measurements were performed 3–5 times for each supernatant.

The ratio of mass fractions of collagen and ChS in cartilage samples was designated as *K*chem_._

#### Determination of collagen content

An acid hydrolyzate of dried cartilage samples weighing 2–5 mg was prepared in a mixture of hydrochloric and trifluoroacetic acids in sealed glass ampoules at a temperature of 166°C. The amino acid analysis was performed on a Hitachi-835 analyzer (Hitachi, Japan) in the standard mode for the analysis of protein hydrolysates. For each hydrolyzate, measurements were performed 3 times. The content of collagen was estimated assuming that hydroxyproline is 13.5% (mass) in the amino acid pool of collagen.

#### Acquisition and processing of IR spectra

The sample spectra were recorded using a Nicolet iS10 infrared Fourier spectrometer (Thermo Fisher Scientific, USA) by the ATR method using a special accessory with a diamond prism. The IR spectra were collected in the reflection mode in the range of 400–4000 cm^–1^ with a resolution of 0.5 cm^–1^ and averaged over 32 scans, converted to log (1/*R*) units using the OMNIC software algorithm. The value of log (1/*R*) is similar to absorbance, since *R* is the ratio of the intensity of infrared energy reflected from the sample to that from a diamond prism without a sample.

IR spectra in the range of 750–1800 cm^–1^ were processed using the TQ Analyst software (Thermo Fisher Scientific, USA).

#### Statistical data processing

The experimental data of chemical analysis and spectral analysis of several samples of the same type were processed using the OriginPro 2015 software package (OriginLab Corporation, USA). The data were presented as М±SD, where М is the mean value and SD is the standard deviation. The difference between the mean *K*_sp_ values for articular cartilage samples taken from visually smooth areas and from rough areas with pronounced fibrousness was assessed by a two-sample Student’s t-test. The *a priori* significance level was set as p=0.05.

The experimental data of spectral analysis using the calibration method by principal component regression were processed using the TQ Analyst software, which provided the root-mean-square errors of calibration, prediction [21], and correlation coefficients.

## Results

### Pure components and calibration mixtures

**Figure 1.**
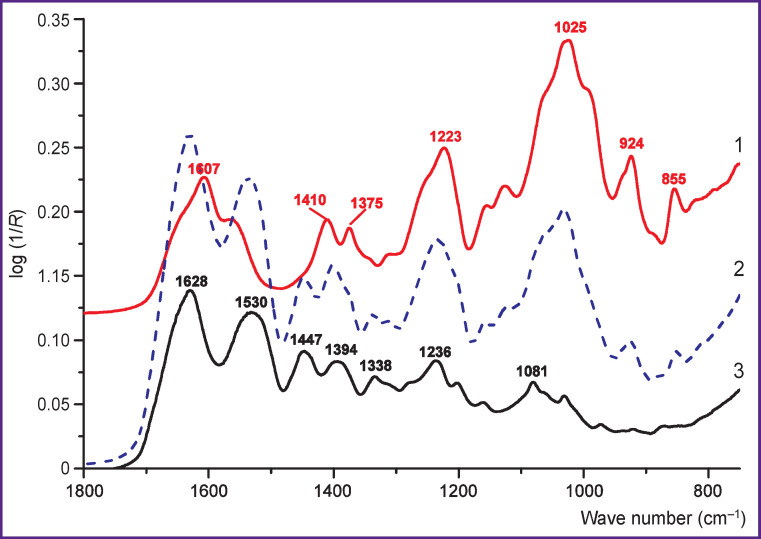
IR spectra of collagen (*curve 3*) and chondroitin-4-sulfate (*curve 1*) and their mixtures with *K*=2.33 (*curve 2*)

The IR spectra of pure components (collagen and ChS) are presented in Figure 1. The IR spectrum of a film collected from a mixture of 70% of collagen and 30% of ChS (by mass) with a *K*_chem_ ratio of 2.33 is also shown there.

The following bands are most characteristic for the collagen IR spectrum:

intense bands with maxima ~1628 and ~1530 cm^–1^ and less intense bands with a maximum of ~1236 cm^–1^ associated with vibrations in the peptide group (Amide I and Amide II bands);

bands with maxima ~1447 and ~1394 cm^–1^ associated with bending vibrations of СН^2^ and СН^3^ groups;

the band representative for collagen with a maximum of ~1338 cm^–1^, presumably associated with the bending vibration of the СН^2^ group in the proline residue in the polypeptide chain.

The following bands appear in the IR spectrum of ChS:

a wide band, in the region of 1650–1500 cm^–1^, associated with vibrations in the N-acetyl group (close to the peptide group by chemical structure) and C=O stretching vibrations in the COO^–^ group;

bands centered at ~1410 and ~1375 cm^–1^, associated with bending vibrations of CH^2^ and CH^3^ or COO^–^ groups;

intense bands centered at ~1223 and ~1025 cm^–1^, associated with symmetric and antisymmetric stretching vibrations in the SO^3–^ group and, possibly, with C-O-C skeletal vibrations (~1025 cm^–1^);

a band centered at ~1125 cm^–1^, associated with the pyranose ring;

bands centered at ~924 and ~855 cm^–1^, associated with skeletal vibrations of С-О-С and С-О-S groups.

**Figure 2.**
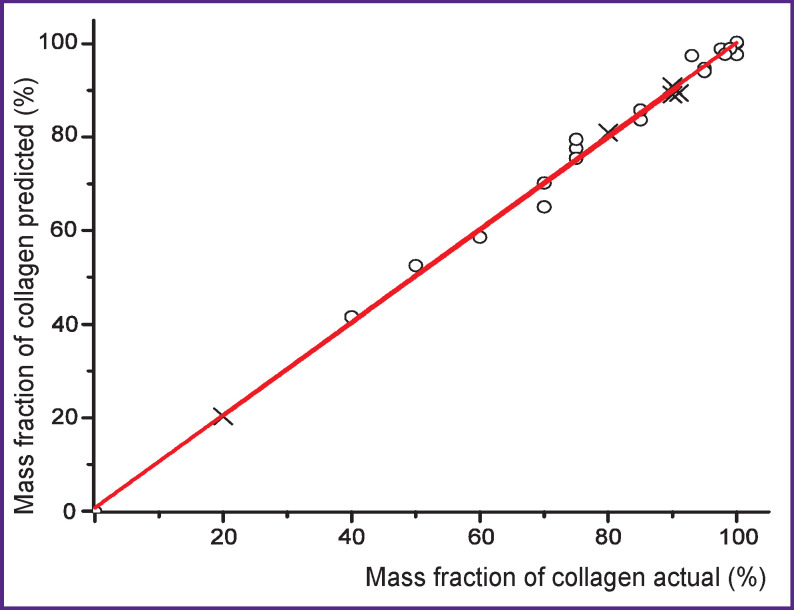
Calibration and verification of a collagen and chondroitin-4-sulfate mixture: ○— a calibration set; ×— a test set with a known composition

Figure 2 shows the calibration dependence for the content of collagen in the collagen–ChS mixture, built using the principal component regression method. The calibration and validation sets (films with a known composition) consisted of 20 and 5 objects, respectively. The root-mean-square errors of calibration and predicting are about 1.9 and 2.3%, and the correlation coefficients are close to 1 both for modeling (*R*^с2^=0.997) and predicting (*R*_t_^2^=0.9978). The calibration and test lines practically merge. Based on calibration dependence, *K*_sp_ was determined as the ratio of the mass fractions of collagen and ChS.

### Cartilage of the nasal septum and its modification with trypsin

**Figure 3.**
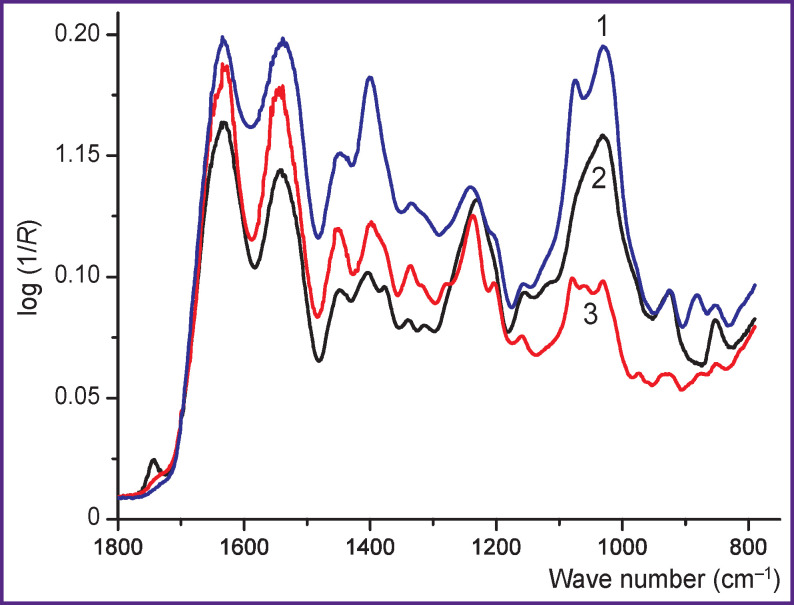
IR spectra of the original cartilage of the nasal septum (*curve 2*), centrifuged insoluble sediment (O2, *curve 1*), and supernatant (C2, *curve 3*) after double trypsin treatment and heating of the original sample

Table 1 shows the content of the main components and the *K*_chem_ and *K*_sp_ values in a sample of cartilage tissue of the nasal septum. The IR spectrum of the original tissue is shown in Figure 3 (*curve 2*).

**Table 1:** Content of the main components and *K*_chem_ and *K*_sp_ values for the cartilage of the nasal septum (CNS)

Sample	Collagen*	Glycosaminoglycans*	*K*_chem_	*K*_sp_
CNS (for one sample)	36.0±0.7	46.0±3.0	0.78	0.81
CNS (n=11)	—	—	—	0.88±0.05

* dry weight percentage.

After the primary trypsinization of the sample with *K*_sp_=0.91, the spectral analysis showed that there was a sharp increase in the proportion of collagen in the insoluble O1 residue (*K*_sp_=4.55) and a decrease in this value in the C1 supernatant film (*K*_sp_=0.32). Nevertheless, after the first trypsinization, a noticeable part of the initial amount of ChS remained in the insoluble O1 residue. After trypsinization, denaturation, and repeated trypsinization of the sample with *K*_sp_=0.89, the vast majority of ChS was found in the C2 supernatant solution (*K*_sp_=0.25), while the insoluble O2 residue contained predominantly collagen (*K*_sp_=13.5). The IR spectra of the O2 centrifugate (Figure 3, *curve 1*) and the C2 film (Figure 3, *curve 3*) differ markedly and approach the IR spectra of collagen and ChS, respectively.

Table 1 also shows the *K*_sp_ value averaged over 11 nasal septal cartilage samples.

### Costal cartilage

It should be noted that the IR spectra of specimens of hyaline cartilage (articular and costal) did not fundamentally differ from those for nasal septum cartilage. Table 2 shows the content of the main components, as well as the *K*_chem_ and *K*_sp_ values for two samples of costal cartilage.

**Table 2:** Content of the main components and *K*_chem_ and *K*_sp_ values for costal cartilage (CC)

Sample	Collagen*	Glycosaminoglycans*	*K*_chem_	*K*_sp_
CC1	29.0±0.5	21.9±2.2	1.32	1.59
CC2	31.0±0.5	18.3±2.1	1.69	1.88

* dry weight percentage.

### Articular cartilage

Articular cartilage samples were taken from both the smooth and rough fibrous surfaces of the joint. Table 3 shows the content of the main components and the *K*_chem_ and *K*_sp_ values for articular cartilage samples with different textures (two samples of each texture). The Table also shows the *K*_sp_ value averaged for 12 samples from a smooth surface and 8 samples from a rough fibrous surface of the joint.

**Table 3:** Content of the main components and *K*_chem_ and *K*_sp_ values for specimens of articular cartilage derived from smooth (ACS) and rough fibrous (ACRF) surfaces

Sample	Collagen*	Glycosaminoglycans*	*K*_chem_	*K*_sp_
ACS-1	60.5±0.7	25.4±2.1	2.38	2.21
ACS-2	58.2±0.5	18.1±1.9	3.21	3.05
ACS (n=12)	—	—	—	2.8±1.1**
ACRF-1	65.1±0.6	12.5±2.5	5.2	5.35
ACRF-2	75.0±0.7	13.9±3.2	5.4	5.7
ACRF (n=8)	—	—	—	4.4±1.8**

* dry weight percentage; ** the mean values of ACS and ACRF were statistically significantly different.

### Nucleus pulposus of the intervertebral disc

**Figure 4.**
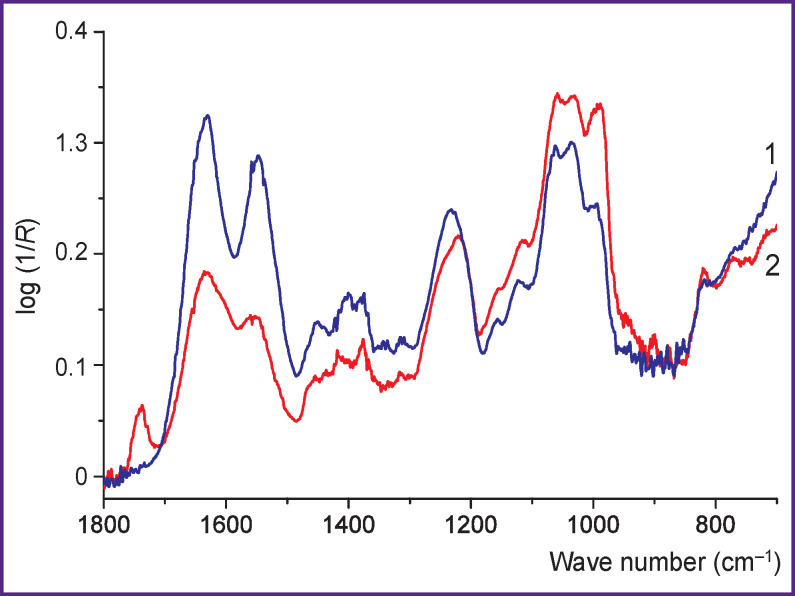
IR spectra of the nucleus pulposus of the intervertebral disc of sheep (*1*) and rabbit (*2*)

Figure 4 shows the IR spectra of specimens of the nucleus pulposus of the intervertebral disc in rabbit and sheep. Table 4 shows the content of the main components and the *K*_chem_ and *K*_sp_ values for different samples of the nucleus pulposus of rabbit and sheep.

**Table 4:** Content of the main components and *K*_chem_ and *K*_sp_ values for the nucleus pulposus of rabbit (PN_rab_) and sheep (PN_sh_)

Sample	Collagen*	Glycosaminoglycans*	*K*_chem_	*K*_sp_
PNrab1	4.9±0.1	28.5±1.5	0.17	0.16
PNrab2	5.8±0.2	31.3±1.7	0.19	0.22
PNsh1	18.1±0.3	13.5±2.1	1.34	1.15
PNsh2	17.2±0.2	12.2±1.8	1.41	1.21

* dry weight percentage.

## Discussion

The main absorption bands in the IR spectra of collagen and GAGs are associated with characteristic vibrations of specific groups in proteins [12, 18, 22, 23] and sulfated amino sugars [18, 24, 25]. The IR spectra of films (Figure 1, *curve 3*) and cartilage tissues (see Figures 3, 4) are a superposition of the IR spectra of collagen and GAGs, which qualitatively reflects their composition. Thus, with an increase in the GAG content, the relative intensity of the bands in the region 980– 1100 cm^–1^ associated with vibrations in the pyranose ring and the SO_^3^_^–^ group increases, and the intensity of the Amide I and Amide II bands of the peptide groups decreases.

Changes in the ratios between the characteristic bands of compounds in the IR spectrum are evident with a significant change in the content of these compounds in cartilage. For example, when comparing the IR spectra of rabbit nucleus pulposus (*K*_sp_~0.2) and sheep nucleus pulposus (*K*_sp_~1.2) shown in Figure 4, an increase in absorption associated with fluctuations in characteristic GAG groups in the first case is clearly noticeable. The manifestation of this effect is also observed during trypsinization, which leads to the destruction of the core and proteoglycan-binding proteins and their aggregates and thus ensures GAG release into the supernatant [26]. In the IR spectra of the solid residue, the GAG absorption bands are far less pronounced than in the spectra of the supernatant (see Figure 2).

A more accurate quantitative characterization of the tissue composition can be obtained only by using calibration models, and preferably with a complete analysis of the IR spectrum, rather than a single ratio of band intensities [11, 12]. It is appropriate to note here that quantitative analysis indicates the need for repeated trypsinization after heating the samples in order to transfer the overwhelming majority of GAGs into the supernatant. Only after such treatment, GAGs are fully available for chemical analysis.

The chemical composition of cartilage determined by the two methods corresponds to the literature data [4–7, 27–29]. This is true both for hyaline cartilage, where *K*_chem_ varies from ~1 in the nasal septum of cattle [27] to ~3–5 in articular cartilage [30], and for the nucleus pulposus (not related to the hyaline type). In the latter case, the proportion of GAGs may exceed the proportion of collagen by several times, depending on the type of animal, and the *K*_chem_ value and the close *K*_sp_ value for the nucleus pulposus of the intervertebral disc of rabbit and sheep, that we have obtained, are in excellent agreement with the data of other authors ([28] and [29], respectively). It should be noted that the same type of cartilage tissue has some variations in the collagen/GAG ratio. This is illustrated by the spread of *K*_sp_ values for several samples from one object (articular cartilage, see Table 2) or several objects (nasal septa, see Table 1). Variations can be associated both with zonal changes in the chemical composition in the integral system and with individual features of a biological object [4–7, 27–30].

The data of spectral IR analysis represent the specifics of articular cartilage degeneration [4–7, 18]. A decrease in the GAG content is an important indicator of this degeneration. We have confirmed that the *K*_sp_ value is significantly higher in cartilage specimens with a rough surface and pronounced fibrillation compared to externally smooth specimens (see Table 2). One should notice that this is true both for the samples in which *K*_chem_ was additionally determined, and for the averaged values of *K*_sp_.

An important result is the coincidence of the *K*_chem_ and *K*_sp_ values for specimens of all types of cartilage, while the ratios of mass fractions of collagen are in a wide range of values. Such a coincidence of these values, one of which (*K*_sp_) was obtained on the whole sample, and the other one — as the amount of the dyed complex of GAGs with DMMB in trypsinate, requires explanation. Let us assume that the error in estimating the content of collagen determined by the most accurate of the analytical methods used is minimal. At the same time, the constants of binding GAG and DMMB dye into a complex for different GAGs differ and the calibration curves for them do not coincide [31], while the calibration dependence used by the researchers is obtained for a specific standard. Thus, one cannot be sure of the exact determination of the total content of all GAGs by measuring the absorption in the supernatant of cartilage tissue after adding a dye, since different GAGs are present in cartilage tissue, their ratios being different, too [32].

There are other factors that stipulate the inaccuracy of the method for determining GAGs using DMMB [33], but this method is still considered to estimate most adequately the “absolute” content of GAGs. As for the IR spectra of different GAGs, there are no fundamental differences in them, since they have the same chemical structures that determine the characteristic bands in the IR spectrum. The controversial moment of the spectral determination of the *K* value is the difference in the composition of calibration mixtures and tissue specimens. However, the similarity of their IR spectra suggests that other components (other than proteins and GAGs) have little effect on the type of the IR spectrum of cartilage tissues. Once again, we underline that we estimate not the absolute amount of collagen and GAGs, but only their ratio, which coincides with the value of the coefficient determined on the basis of chemical analysis.

Experimental determination of a collagen/GAG ratio based on the IR spectrum (*K*_sp_) obtained by employing ATR does not require multi-stage sample preparation, which is costly, time-consuming, and requires highly skilled analysis performers. In addition, the chemical determination of collagen and GAG cannot be done in one and the same sample, and the analyzed samples may not be identical. Knowing the spectral ratio in the sample, it is possible to select and conduct an analysis of one of the components that is more convenient for the researcher. The amount of the second component is easily calculated. Determination of the *K*_chem_ value is often the first one when studying cartilage tissue changes, and the number of samples can amount to several tens. In this case, the express characterization of the variability of the chemical composition is extremely important.

## Conclusion

Infrared spectrometric analysis in the mode of attenuated total reflection employing the method of principal component regression makes it possible to determine correctly the ratio of collagen and glycosaminoglycans, the main components of the cartilage matrix, and to conduct a preliminary non-invasive diagnosis of cartilage tissue. We have proposed an express method to determine the features and changes in the composition for a large set of cartilage tissue samples of several types. An additional advantage is an extremely simple sample preparation, which does not require multi-stage and time-consuming chemical manipulations and the reagent consumption.
